# A 20-year real-world study on drug survival and predictive factors for infliximab discontinuation in Behçet’s syndrome

**DOI:** 10.3389/fimmu.2025.1726690

**Published:** 2025-12-08

**Authors:** Rosaria Talarico, Antonello Sulis, Federica Di Cianni, Diana Marinello, Maria Laura Manca, Marta Mosca

**Affiliations:** 1 RheumatologyUnit, Azienda Ospedaliero-Universitaria Pisana, Department of Clinical and Experimental Medicine, University of Pisa, Pisa, Italy; 2 Department of Medical Biotechnologies, University of Siena, Siena, Italy; 3 Department of Clinical and Experimental Medicine and Department of Mathematics, University of Pisa, Pisa, Italy

**Keywords:** Behçet’s syndrome, anti TNF alpha agents, infliximab, drug survival, retention on treatment

## Abstract

**Objective:**

Infliximab (IFX) is an immunosuppressive drug widely used for the treatment of patients with Behçet’s syndrome (BS) with severe or refractory organ involvements. The aim of this study was to evaluate IFX survival, clinical response and safety profile in a monocentric cohort of BS patients.

**Methods:**

Patients with BS treated with IFX intravenously across a 20-year period were retrospectively-prospectively examined. Total duration of therapy and drug retention until the end of follow-up were calculated, as well as the reasons of discontinuation, including adverse events. Clinical response, expressed as changes in BDCAF (Behçet’s Disease Current Activity Form) indexacross the follow-up period, were evaluated and compared among patients.

**Results:**

Sixty patients with BS were treated with IFX over a 20-year period. Overall drug retention was 45%, with survival rates of 78.3% at 1 year, 63.3% at 2 years, and 35% at 5 years (median duration 37 months, IQR 17–72). Most discontinuations occurred after 24 months and were mainly due to loss of efficacy (25%), de novo manifestations (21%), or allergic reactions (18%). Clinical activity improved markedly, with a mean BDCAF reduction of 4 points, ranging from −3.0 in mild to −5.7 in highly active disease. Continuers showed lower BDCAF scores at last follow-up and achieved higher remission and major response rates compared with discontinuers. Younger age and female sex were associated with higher discontinuation risk. Over the two decades, IFX indications shifted from major-organ involvement to predominantly refractory muco-cutaneous disease.

**Conclusions:**

IFX showed good safety profile and excellent clinical response in this real-world BS cohort, with a drug survival of 45% within a median treatment duration of 37 months.

## Introduction

Behçet’s syndrome (BS) presents with a broad spectrum of clinical manifestations with milder forms, mainly characterized by muco-cutaneous lesions and joint involvement, and more severe disease phenotypes, with ocular inflammation, central nervous system (CNS) and vascular involvement (1, 2). The multi-systemic nature of BS, combined with its unpredictable and relapsing course, often complicates disease management and leads to development of irreversible organ damage (3–5). Several demographic variables, including younger age at disease onset, male sex, and longer disease duration, have been linked to worse clinical outcomes and increased risk of complications in both the short and long term (6, 7); in particular, younger age at disease onset and male sex, have been linked with a more aggressive disease course and an increased risk of long-term complications, morbidity, and mortality in BS (2, 8, 9). Overall, BS has a considerable negative impact on health-related quality of life, influenced by both the severity and chronicity of symptoms (10–12). Among immunological pathways implicated in the pathogenesis (13, 14), tumour necrosis factor-alpha (TNF-α) plays a central role in driving the inflammatory response, contributing to endothelial dysfunction and tissue damage observed in the disease (15, 16). This has led to the off-label use of TNF-α inhibitors, particularly infliximab (IFX) and adalimumab (ADA), for severe or refractory disease phenotypes. IFX is a chimeric monoclonal antibody directed against TNF-α and was originally approved for the treatment of rheumatoid arthritis, Crohn’s disease, and other inflammatory disorders. Since the early 2000s, its use has expanded to BS, especially for patients with sight-threatening uveitis, and neuro-Behçet’s disease (NBD), where traditional immunosuppressants such as azathioprine or cyclosporine have failed to achieve adequate control (17, 18). Observational studies have consistently shown IFX to induce rapid symptom resolution, reduce the frequency and severity of flares, and preserve organ function, particularly visual acuity in uveitis patients (19–24). Recently, we have published a multicentre randomised controlled study (RCT) directly studying IFX and ADA in patients with active, refractory BS, and demonstrating that both agents were effective in reducing disease activity and achieving sustained remission, with no significant differences in primary efficacy outcomes. However, slight differences were observed in secondary outcomes, including adverse events and patient-reported tolerability, highlighting the need for individualized treatment decisions (25).

However, even with its proven clinical benefits, several challenges remain in the long-term management of BS with IFX. One of the key questions pertains to treatment persistence, commonly referred to as drug survival, which is a composite endpoint reflecting the balance among efficacy, safety, tolerability, and patient satisfaction in real-life clinical settings. High drug survival implies sustained benefit with an acceptable safety profile, while premature discontinuation may indicate a loss of efficacy, adverse events, remission, or logistical issues such as access to therapy or drug availability (23, 24). Despite the growing clinical use of IFX, systematic data on its long-term use in real-world populations with BS remain scarce. Most existing studies are focused on specific phenotypes (e.g., ocular BS), or include small sample sizes that limit the generalisability of their findings (9, 26). Furthermore, real-world evidence on the reasons for treatment discontinuation, such as loss of efficacy, infusion reactions, or remission, is still lacking. In addition, the relationship between treatment duration and longitudinal disease activity, which may guide therapeutic decisions on continuation or withdrawal, has not been fully explored. Accordingly, the European Alliance of Associations for Rheumatology (EULAR) recommendations for the management of BS, indicate that monoclonal anti-TNF-α antibodies should be considered in severe disease as first-line or in refractory patients (27). By capturing longitudinal clinical outcomes in a real-world setting, this study aims to provide meaningful evidence to inform long-term therapeutic strategies in BS.

## Methods

### Study design and population

We conducted a retrospective-prospective cohort study of patients aged18 ≥ years with a confirmed diagnosis of BS according to International Study Group criteria (28), who initiated IFX in our centre between 2004 and 2024.Patients received IFX 5 mg/kg as an intravenous infusion at weeks 0, 2, and 6,and at 6–8 week-intervals thereafter in the outpatient infusion clinic of our centre.

Demographics, disease duration, previous organ involvements of disease, reasons for initiating IFX, duration of follow up and previous and concurrent medications were recorded. This study comprised a retrospective phase (2004–2018) and a prospective phase starting in 2019. Retrospective data were extracted from electronic health records and infusion clinic registries, which routinely included BDCAF scores, infusion dates, adverse events, organ involvement, and treatment modifications documented at each visit. From 2019 onward, the same variables were collected prospectively using a standardized data-collection protocol applied at every scheduled or unscheduled visit. Data quality in the prospective phase was ensured through predefined electronic templates.

The study was conducted in accordance with the Declaration of Helsinki and received approval from the Tuscany Regional Ethics Committee – Paediatric Division (CERT-P), protocol code n. 122/2024, approved on 09/07/2024.

### Study outcomes and follow-up

The main objective of the study was to address these knowledge gaps by evaluating the drug survival of IFX in a real-life cohort of patients with BS, identifying the primary reasons for treatment discontinuation, and examining changes in disease activity over time.

Disease activity - measured according to the Behçet’s Disease clinical activity form index (BDCAF)- and outcomes were recorded at IFX initiation and at regular follow-up visits, until drug discontinuation or until the end of follow-up (December 31, 2024), whichever came first. More specifically, BDCAF scores were assessed at baseline and at every IFX infusion, which were routinely scheduled every 6–8 weeks. BDCAF was also recorded at unscheduled visits if patients presented with new or worsening symptoms, ensuring consistent longitudinal monitoring.

The primary outcome was discontinuation of IFX therapy, for any cause. Retention rates and the median duration of treatment were also calculated. Data on reasons for discontinuation were collected as reported by the treating rheumatologist for the following pre-specified categories: loss of efficacy (relapse of the disease after at least 6 months of treatment), inefficacy (failure to achieve remission or relapse after ≤ 6 months), occurrence of *de novo* manifestations (the emergence of new BS manifestations that occurred for the first time during treatment with IFX), allergic reactions, recurrent infections, other adverse events (including infusion reactions or events other than recurrent infections or allergic reactions), death and pregnancy. Patients were assumed lost to follow-up when they had missed drug infusions for ≥ 4 months. *De novo* manifestations were defined as new BS manifestations appearing for the first time during IFX treatment in patients who had never shown those specific manifestations before initiation. Based on clinical assessment, these events were classified as disease flares in a previously uninvolved organ system rather than paradoxical inflammatory reactions to anti-TNF therapy. No manifestations were considered unrelated incidental findings. This distinction was made by the treating rheumatologist after multidisciplinary evaluation, considering timing relative to treatment, inflammatory characteristics, and exclusion of alternative causes.

Secondary outcomes included clinical response and safety profile of IFX, as well as demographic and clinical predictors of treatment discontinuation (including gender, age and organ involvement). Clinical response to IFX was assessed by analysing BDCAF index and the changes in BDCAF index from the treatment initiation to the last follow-up across the entire cohort. The following definitions were used: clinical remission as a BDCAF index ≤ 2, a major clinical response as ≥50% reduction in BDCAF index, improvement as a reduction in BDCA Findex by 1 point and worsening as an increase in BDCAF index by 1 point. Clinical response was also compared among continuers (patients still receiving IFX at the end of follow-up) and discontinuers (patients who had suspended treatment before the end of follow-up).

Additional secondary outcomes included the therapeutic switching patterns following IFX discontinuation, and the calendar period changes in IFX prescription across the 2004–2024 period.

### Statistical analysis

All statistical analyses were performed using R software (version 4.5.1). Normality of continuous variables was assessed using the Shapiro-Wilk test. Due to non-normal distribution of most clinical variables, continuous data are reported as median (interquartile range, IQR) and compared between groups using the Mann-Whitney U test. Categorical variables are presented as absolute numbers and percentages [n (%)] and compared using chi-square test or Fisher’s exact test when appropriate (expected cell count <5). Complete case analysis was performed for all statistical comparisons. Follow-up duration was calculated from IFX initiation to last clinical assessment or until study endpoint. Univariate logistic regression was performed to identify factors associated with discontinuation of IFX therapy. Variables with p<0.20 in univariate analysis were included in multivariate logistic regression models. Results are presented as odds ratios (OR) with 95% confidence intervals (CI). Multiple comparison correction was applied using the Bonferroni method where appropriate. A two-sided p-value <0.05 was considered statistically significant.

### Patient and public involvement

Patients were not involved in the study.

## Results

### Patients’ characteristics

A total of 60 BS patients treated with IFX between 2004 and 2024were identified. Patients were mainly women (56.7%),and the mean age at IFX introduction was 41.6 years. The mean delay between disease onset and IFX introduction was 10.4 years. **Table 1** summarizes patient characteristics, including prior organ involvement requiring IFX introduction and previous medications.

**Table 1:** Patients’ characteristics at IFX introduction.

Number of patients	N=60
Sex, N (%)	
Men	26 (43.3%)
Women	34 (56.7%)
Age at diagnosis (median, IQR)	31 years (21.5)
Age (median, IQR)	41.6 years (15)
Delay of IFX introduction (median, IQR)	7 years (11)
Follow-up (mean, range)	10 years (3-22)
Previous organ involvements (%)	
Oral aphthosis	43 (72)
Genital aphthosis	21 (35)
Skin lesions	21 (35)
Arthritis	14 (23)
Gastrointestinal manifestations	14 (23)
Ocular involvement	17 (28)
CNS involvement	9 (15)
Unknown	2 (3)
Previous therapies N (%)	
ADA	15 (25)
GOL	2 (3)
CTZ	2 (3)
ETN	2 (3)
ANAK	1 (2)
APR	2(3)
AZA	11 (18)
MTX	10 (17)
CYA	8 (13)
Colchicine	2 (3)
CFX	2 (3)
None	14 (23)

ADA, adalimumab; ANAK, anakinra; APR, apremilast; AZA, azathioprine; CFX, cyclophosphamide; CTZ, certolizumab; CYA, cyclosporine; ETN, etanercept; GOL, golimumab; IFX, infliximab; MTX, methotrexate.

### Retention on treatment and safety profile

During the follow up, the median duration of treatment was 37 months (IQR 17-72) and overall retention on treatment was 45%. The retention on treatment was 78.3% at 1 year, 63.3% at 2 years and 35% at 5 years. IFX suspension mainly occurred after 24 months (57.6%). The most common reasons for discontinuation over the full period were loss of efficacy (25%, 8/33), *de novo* manifestations (21%, 7/33), and allergy (18%, 6/33). Additional discontinuation reasons and discontinuation timing are indicated in **Table 2**. The timing of discontinuation showed distinct patterns across causes. Allergic reactions occurred early, typically within the first 6–12 months of treatment, whereas loss of efficacy and *de novo* manifestations emerged later, predominantly after more than 24 months of IFX therapy.

**Table 2:** Retention on drug, discontinuation timing, discontinuation reasons and therapeutic switches after drug discontinuation.

Number of patients	Total patients, N = 60
Retention on treatment (%)	
Overall	33 (55)
At 1 year	47 (78.3)
At 2 years	38 (63.3)
At 5 years	21 (35)
Duration of treatment (median)	37 months (IQR 17-72)
	**Discontinuers N = 33**
Discontinuation timing (%):	
≤ 6 months	2 (6.1)
6–12 months	5 (15.2)
12–24 months	7 (21.2)
over 24 months	19 (57.6)
Discontinuation reasons (%)	
Allergy	6 (18)
Severe allergy	2 (6)
Loss of efficacy	8 (24)
Inefficacy	1 (3)
*De novo* manifestations	7 (21)
Other adverse reactions	3 (10)
Recurrent infections	2 (6)
Pregnancy	4 (12)
Therapeutic switches post-IFX:	
ADA	8 (24)
GOL	6 (18)
CTZ	4 (12)
AZA	5 (15)
Others	8 (24)
No further treatment	4 (12)

ADA, adalimumab; AZA, azathioprine; CTZ, certolizumab; GOL golimumab; IFX infliximab.

Discontinuers N = 33 is indicating the number of patients who discontinued the therapy.

After IFX, discontinuers mainly switched to adalimumab (ADA, 24%), followed by golimumab (GOL, 18%) and certolizumab (CTZ, 12%).

### Drug clinical response

Analysis of BDCAF index across the entire cohort showed an average reduction of 4 points at the end of the follow-up. The reduction was greater in case of higher disease activity at IFX initiation, in particular BDCAF index reduced by 3 points in patients with mild disease activity (BDCAF ≤4) at IFX introduction, by 3.7 points in patients with previous moderate activity (BDCAF 5-7) and by 5.7 points in patients with high activity (BDCAF >7).When comparing continuers and discontinuers, we found that BDCAF index at baseline was similar in the two groups (5.9 in continuers vs 6.4 in discontinuers), whereas the index at last follow-up(namely, the end of follow-up for continuers and the follow-up visit at IFX suspension for discontinuers) was borderline significantly lower in continuers. We also found that continuers achieved higher major response (≥ 50% reduction in BDCAF index) and clinical remission(BDCAF index ≤ 2) at the end of follow-up compared to discontinuers. No patient showed a worsening of BDCAF index at the end of follow up. To account for baseline BDCAF differences, we also evaluated categorical, baseline-adjusted outcomes. Major response (defined as ≥50% reduction in BDCAF from baseline) and clinical remission (BDCAF ≤2) were more frequently achieved among continuers compared with discontinuers. These categorical measures provide a baseline-adjusted interpretation of clinical improvement that is less dependent on initial disease activity. Details on BDCAF changes and comparison among discontinuers and continuers are provided in **Table 3**.

**Table 3:** Overall clinical response variation refers to BDCAF changes from IFX initiation to the end of follow up.

Disease activity category	BDCAF variation
BDCAF reduction (mean)	- 4 points
Clinical response according to basal disease activity	
Mild activity (BDCAF ≤4),Δ variation	Δ= - 3.0 points
Moderate activity (BDCAF 5-7),Δ variation	Δ= - 3.7 points
High activity (BDCAF >7),Δ variation	Δ= - 5.7 points

BDCAF, Behçet’s disease current activity form.

### Predictors of discontinuation

Female gender (Male 34.6%, Female 70.6% p-value <0.01, OR 4.53 (IC95% 0.84-7.69)) and younger age (< 40 years 68%, ≥ 40 years 45.7%) at IFX introduction were significantly associated with a higher risk of discontinuation. In contrast, specific disease organ involvement did not significantly predict drug discontinuation.

### Calendar period changes of indication for IFX

The clinical characteristics of patients receiving IFX across 2004–2024 are shown in **Table 4**.

**Table 4:** Characteristics of patients treated with IFX, stratified for period.

Period	Patients N (%)	Major involvement* N (%)	CNS N (%)	Ocular N (%)	Muco-cutaneous N (%)	Articular N (%)
1^st^period: 2004-2012	12 (20)	6 (50.0)	3 (25.0)	5 (41.7)	7 (58.3)	2 (16.7)
2004-2006	3 (5.0)	2 (66.7)	1 (33.3)	1 (33.3)	2 (66.7)	0 (0.0)
2007-2009	2 (3.3)	1 (50.0)	1 (50.0)	0 (0.0)	1 (50.0)	0 (0.0)
2010-2012	7 (11.7)	3 (42.9)	1 (14.3)	4 (57.1)	4 (57.1)	2 (28.6)
2^nd^period: 2013-2018	11 (18.3)	4 (36.4)	2 (18.2)	2 (18.2)	9 (81.8)	2 (18.2)
2013-2015	6 (10.0)	3 (50.0)	2 (33.3)	2 (33.3)	5 (83.3)	0 (0.0)
2016-2018	5 (8.3)	1 (20.0)	0 (0.0)	1 (20.0)	4 (80.0)	2 (40.0)
3^rd^period: 2019-2024	37 (61.7)	10 (27.0)	4 (10.8)	7 (18.9)	34 (91.9)	10 (27.0)
2019-2021	25 (41.7)	6 (24.0)	3 (12.0)	3 (12.0)	23 (92.0)	7 (28.0)
2022-2024	12 (20.0)	4 (33.3)	1 (8.3)	4 (33.3)	11 (91.7)	3 (25.0)
Total:	60 (100.0)	20 (33.3)	9 (15.0)	14 (23.3)	50 (83.3)	14 (23.3)

*Major involvement includes at least one among neurological and ocular.

CNS, central nervous system.

In the first period (2004–2012) 12 patients received IFX, with major organ involvement (CNS or ocular involvement) accounting for 50% of indications. In the second period (2013–2018) 4 out of 11 patients (36%) receiving IFX were treated for major organ involvement. In the most recent period (2019–2024), 37 patients initiated IFX, but only 27% of these showed major organ involvement. Notably, in this latter case, the primary indication for IFX were refractory muco-cutaneous lesions (92%). The overall treatment indications from 2004 to 2024 were refractory muco-cutaneous involvement (83%).

**Figure 1:**
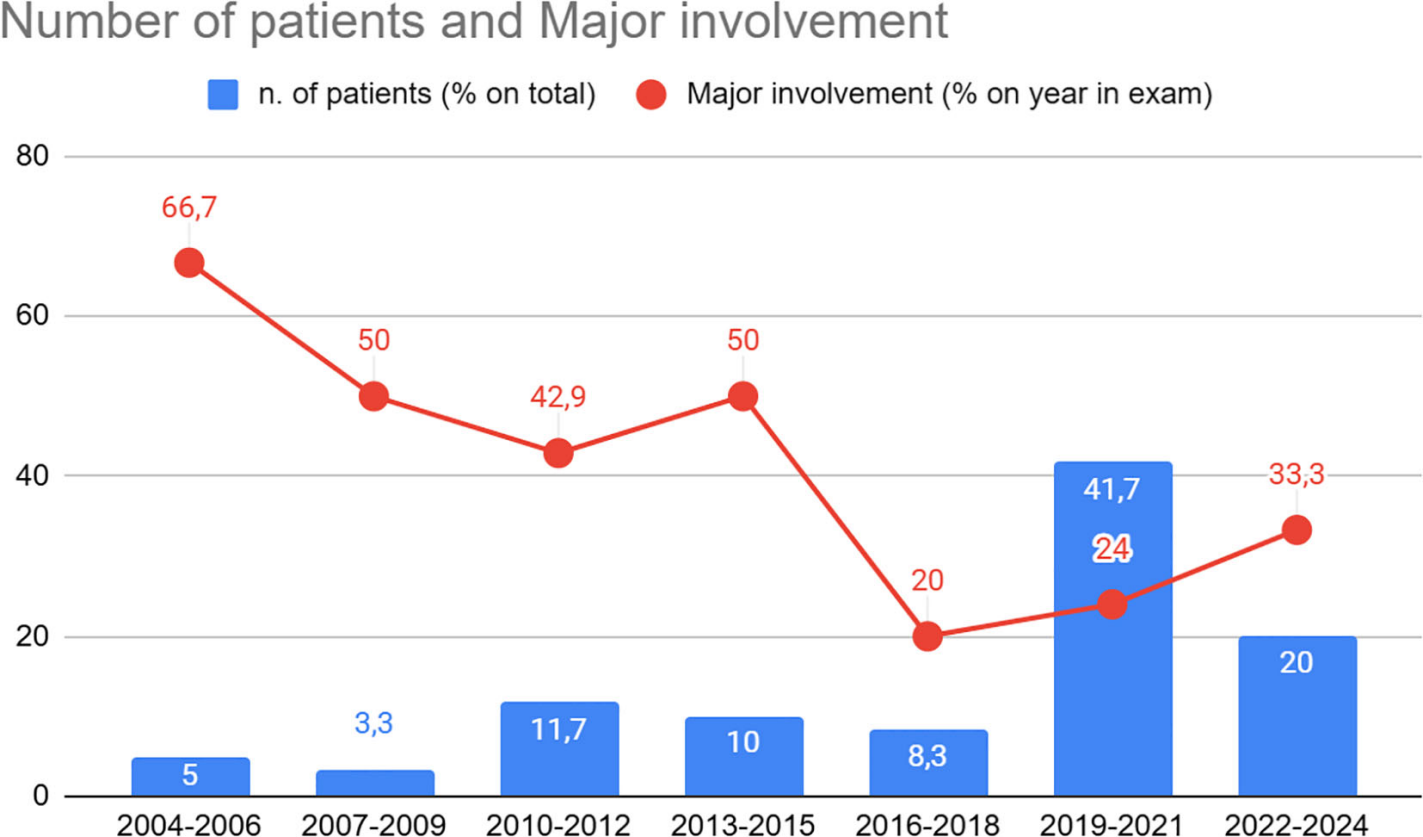
Trend in the treatment indication for IFX, stratified by biennium. Y-axis: percentage of patients receiving IFX in each biennium. The values displayed within the bars indicate the percentage relative to the total number of patients treated. Percentages shown in red represent the proportion of patients with major organ involvement within the same biennium.

**Figure 2:**
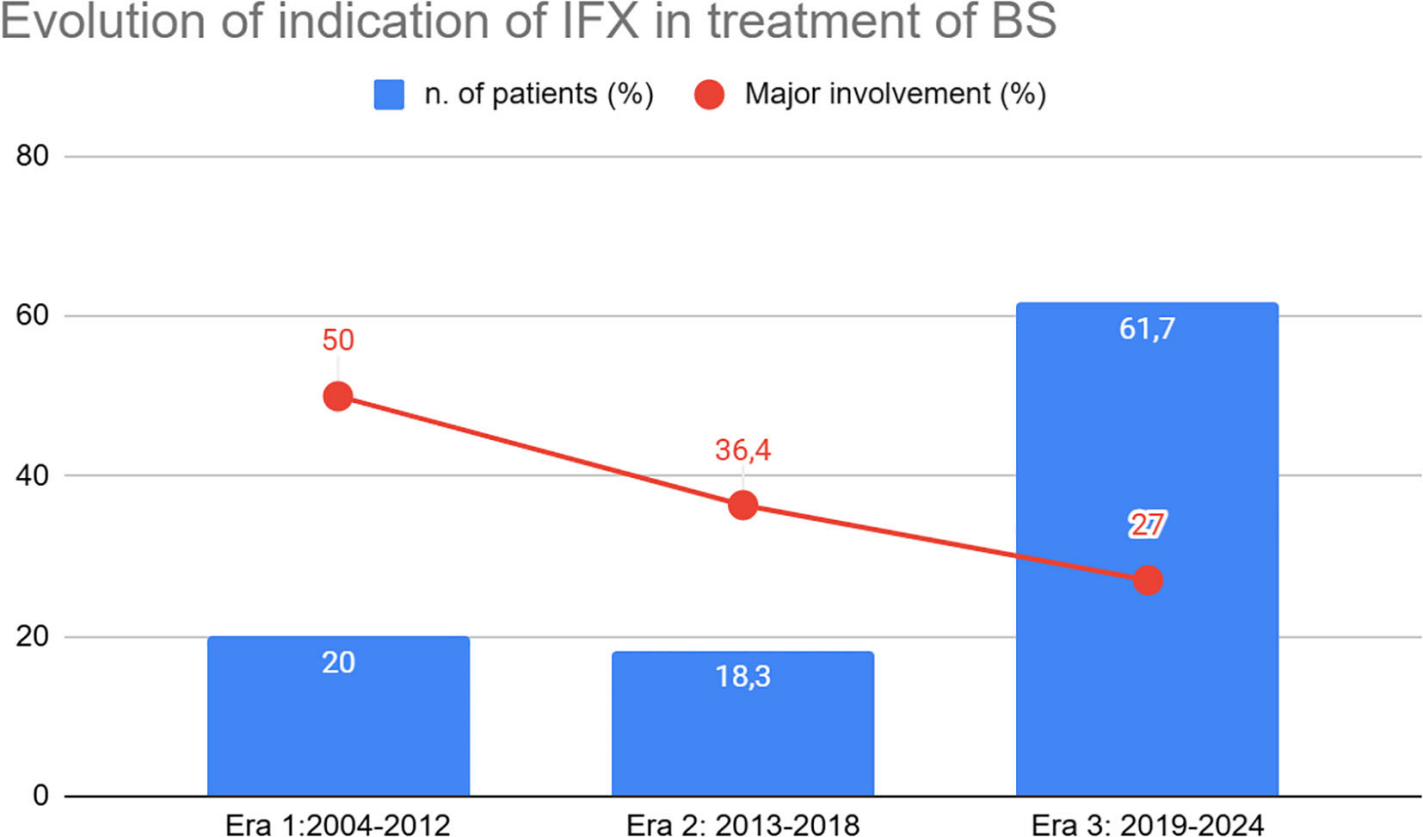
Trend of indication for IFX stratified by eras. Y-axis: percentage of patients receiving IFX in each biennium. The values displayed within the bars indicate the percentage relative to the total number of patients treated. Percentages shown in red represent the proportion of patients with major organ involvement within the same biennium.

Trends in the number of patients receiving IFX and the corresponding treatment indications during the follow-up period are illustrated in **Figures 1** and **2**. The total number of patients receiving IFX increased steadily, from 3 in 2004–2006 to 25 in 2019-2021. On the other hand, the proportions of patients with major organ involvement declined from 67% in 2004–2006 to 33% in 2022-2024, reaching the lowest drop in 2016-2018 (20%).

## Discussion

This real-world study provides comprehensive, long-term data on the use of IFX in BS, focusing on both drug survival and clinical effectiveness over a 20-years period. We found a treatment discontinuation rate of 55%, primarily due to loss of efficacy, allergic reactions, and the emergence of *de novo* disease manifestations. Notably, the drug demonstrated a favourable safety profile, with only two cases of anaphylaxis and two of recurrent infections recorded; the remaining adverse events were predominantly mild, self-limiting, or infusion-related reactions. Several authors previously observed few or no serious adverse events in cohort studies, supporting IFX safety and tolerability in real-life (18, 20, 23). As shown in the results, the timing of IFX discontinuation varied substantially across causes. Allergic reactions tended to occur early, usually within the first months of therapy, whereas both loss of efficacy and *de novo* manifestations were predominantly observed after more prolonged exposure. This temporal distribution is clinically meaningful: early withdrawals are consistent with immediate or subacute immuno-allergic mechanisms, while late withdrawals more likely reflect either the natural evolution of BS in previously uninvolved domains or a gradual waning of IFX effectiveness over time. These patterns underscore the heterogeneity of discontinuation drivers and highlight the importance of differentiating early adverse reactions from later disease- or treatment-related phenomena when interpreting IFX durability.

At the end of follow-up, 45% of patients remained on IFX, with a median treatment duration of 37 months. Drug survival rates were 78.3% at 1 year, 63.3% at 2 years, and 35% at 5 years. These data are in line with previously published real-life cohorts studies, although some of them report higher retention rates, possibly reflecting differences in population characteristics, treatment indication, or follow-up duration (23). Similarly, an open-label, prospective study reported on IFX long-term efficacy and safety, but study population was smaller, and only ocular outcomes were addressed (20). The high retention observed during the first year likely reflects the favourable short-term efficacy and tolerability of IFX. Early discontinuations were mainly related to allergic phenomena, and patients who tolerated the initial infusions typically experienced rapid symptom improvement and stable disease control, supporting persistence in the first 12 months. This pattern aligns with retention trajectories reported in other real-world anti-TNF cohorts.

Our analysis showed a marked reduction of BDCAF, particularly prominent in patients with a higher BDCAF index at baseline. In addition, patients still receiving IFX at the end of follow-up were more likely to achieve clinical remission and major response. These findings support the strong clinical response of IFX and its potential to induce and maintain long-term remission. Because absolute BDCAF reductions are influenced by baseline disease activity, categorical outcomes such as major response (≥50% improvement) and remission (BDCAF ≤2) offer a more meaningful and baseline-adjusted measure of clinical benefit. In our cohort, these measures confirmed the greater improvement observed in patients who remained on IFX compared with those who discontinued treatment.

In the present study, the discontinuation rate on IFX was 55%. However, it should be noted that all the patients who discontinued the treatment for any reasons were included (e.g. also due to adverse events), which likely contributed to the overall higher discontinuation rate throughout the follow-up period. These temporal patterns suggest different underlying mechanisms for early versus late withdrawals. Early discontinuations were mainly driven by allergic reactions, consistent with immuno-allergic phenomena that typically arise within the first months of therapy. In contrast, later discontinuations—largely due to loss of efficacy or *de novo* manifestations—likely reflect disease-related evolution or gradual waning of treatment response rather than true paradoxical events. From a clinical perspective, the occurrence of *de novo* manifestations during IFX therapy should not be interpreted as paradoxical inflammation in most cases, but rather as part of the natural multi-systemic evolution of BS. These manifestations often involve domains previously unaffected at treatment initiation and may indicate either partial disease control or insufficient suppression of subclinical activity. Their recognition is clinically important, as they frequently prompt treatment escalation or switching. Additionally, the prolonged follow-up may have further influenced the observed discontinuation rate.

We found that female sex significantly predict discontinuation. It might be speculated that this is partially due to the therapeutic switch to CTZ in female pregnant patients. Age at drug introduction was also identified as a statistically significant predictor of discontinuation. Indeed, young patients with BS often show a more severe disease course, which may lead to therapeutic changes in an effort to achieve sustained control of disease. However, these findings should be interpreted with caution. The number of discontinuation events was limited, resulting in wide confidence intervals and reduced statistical power of the multivariable model. For this reason, the associations between female sex, younger age and discontinuation should be considered exploratory rather than definitive predictors. Beyond baseline BDCAF values, continuers and discontinuers differed in additional clinically relevant aspects. Patients who remained on IFX tended to display a more stable disease phenotype over time, fewer infusion-related reactions, and overall better tolerability, all of which may contribute to longer treatment persistence. Conversely, discontinuers were more likely to develop new organ involvement or intolerance prompting therapeutic modification. These factors help explain treatment persistence beyond the magnitude of BDCAF reduction alone. Moreover, in our cohort, the rationale for switching to specific TNF inhibitors followed consistent clinical patterns. Switches to ADA were frequently motivated by pregnancy planning or by the preference for self-administered subcutaneous formulations, which offer greater flexibility than intravenous IFX. In contrast, switches to GOL or CTZ were predominantly driven by intolerance or inadequate response to IFX. These patterns may partially explain the sex-related differences observed in discontinuation and switching behaviour, as female patients more commonly required treatment adjustments related to reproductive considerations and convenience of administration.

Interestingly, the temporal shift in indications for IFX observed in our cohort reflects broader changes in clinical practice over the past two decades. In the early 2000s, IFX was predominantly reserved for severe, sight- or life-threatening manifestations such as ocular or neurological involvement, in line with the limited evidence and reimbursement constraints of that time. Over the years, several factors contributed to a progressive expansion of its use. First, accumulating real-world experience and observational studies supported the efficacy of anti-TNF agents in refractory muco-cutaneous disease (25). Second, the publication of updated EULAR recommendations (27) and the results of RCTs demonstrating the benefit of TNF inhibitors for oral and genital ulcers strengthened clinicians’ confidence in using these drugs earlier in the disease course. Third, improved access to biologics and more flexible reimbursement policies facilitated their prescription beyond major organ involvement. Finally, increased familiarity with IFX and other TNF inhibitors in routine practice promoted a broader therapeutic application across disease spectra. Together, these factors explain the observed transition from treating mostly major-organ disease to predominantly muco-cutaneous involvement in recent years.

This study presents a relatively large cohort, particularly in light of the rarity of the disease, with long-term follow-up and inclusion of patients with multi-organ involvement. Data span the entire period since IFX became available, offering a comprehensive overview of its use. As an observational, single-centre study, it provides insight into routine clinical practice.

Nevertheless, several limitations must be acknowledged. The retrospective design may have introduced missing data and selection bias. Moreover, as this is a single-centre study, the generalizability of these findings to other populations or healthcare settings may be limited.

Additionally, patients were not stratified according to prior exposure to other anti-TNF-α agents or other biologic therapies, and concomitant immunosuppressive treatments were not systematically evaluated. Future studies, ideally prospective and involving larger cohorts, should aim to confirm these findings and explore the efficacy of IFX and other drugs in relation to specific organ involvement, prior or concomitant treatments, and potential predictors of treatment discontinuation. This will contribute to the development of more precise, tailored treatment strategies for BS patients.

## Conclusions

In conclusion, this study confirms the long-term efficacy and safety of IFX in patients living with BS, with high treatment retention rates at two years and a low incidence of serious adverse events. In this real-world setting, IFX was associated with a significant reduction in disease activity, as measured by BDCAF index, further supporting its therapeutic role in BS. Notably, younger age and female sex emerged as independent predictors of treatment discontinuation, a finding that warrants further investigation to clarify their relationship with treatment failure.

The results of our study also support the use of IFX across a broad spectrum of disease phenotypes, including both major organ involvement and refractory muco-cutaneous manifestations, in line with current therapeutic recommendations. These real-world data reinforce the importance of a personalized approach to BS management, enabling clinicians to tailor therapy based on individual patient characteristics. Future prospective studies should confirm these findings and help refine individualized treatment strategies in BS.

## Data Availability

The raw data supporting the conclusions of this article will be made available by the authors, without undue reservation.
